# Medical Opinion and Movement

**Published:** 1909-09-25

**Authors:** 


### Medical Opinion and Movement.

A PRELIMINARY report appears in the *Münchner Medizinischer Wochenschrift* upon some research carried out by H. Conradi, of Neuenkirchen, upon the internal organs of healthy cattle and swine to determine whether or not they are free from bacteria. It is generally supposed that normal liver, spleen, kidney, and blood are entirely free from any microbes. On the other hand the lungs, mesenteric, and other glands may admittedly contain them. Conradi, however, finds bacteria present, although generally in very sparse quantities, in all these organs. He claims, of course, to use a special technique which absolutely excludes any possibility of contamination. Bacteria were found in nearly two-thirds of all the livers examined and in one-third of the kidneys and muscles. Bacteria were also present in four out of five lungs, one out of four lymph glands, and in one of eleven spleens. Anaerobic bacteria were very common. The types of organisms show that the majority belong to the intestinal flora. It is intended to continue these researches, and it seems possible that they may throw light upon the question of auto-infection and the relation between trauma and infection.

DRS. E. WEILL AND G. MOURIQUAND advise the administration of Oxygen as the most efficacious therapeutic measure in severe cases of Whooping Cough. Dr. Weill has used this method of treatment for some years in all cases of broncho-pneumonia in children, and has found more recently that it is equally useful for whooping cough. He claims that it not only hinders the development of broncho-pneumonia, but also rapidly diminishes the violence of the coughing fits and the consequent cyanosis. The cases treated in this way amount to about thirty, and may be grouped into two categories; in the one the intensity of the fits formed the chief danger, and in the other there was added the complication of broncho-pneumonia. Although oxygen inhalations may not actually cure a broncho-pneumonia focus, it is claimed that they act as an antiseptic purifying agent, and so prevent its extension. Moreover, whereas most of the drugs used to allay the intensity of the fits are depressants, oxygen inhalations are stimulating as well as sedative, and act as a tonic to the organism in the struggle with the disease. In cases of simple severe whooping cough the oxygen should be administered immediately on the onset of a coughing fit; but when broncho-pneumonia is threatened or present, it should also be given between the fits, about every hour. The authors are especially emphatic on the necessity of giving the oxygen freely—as much as 10 or 20 litres at the onset of each fit.

DRS. LABBE AND VARET, electro-therapeutists of L'Hôpital de la Charité, advocate the administration of Ozone in the treatment of Whooping Cough. The treatment was first used by Dr. Hellet, of Clichy, in 1890, and it has since been employed and reported upon by several others. Drs. Labbé and Varet have treated eighteen cases since 1907, and a report of their work appears in the *Journal de Médecine*. The patients were brought to the hospital every day or every other day, and received the ozonic inhalations. The duration of the disease under this treatment was from nine to twenty-seven days, or a mean duration of twenty days, although most of the cases were of moderate severity, and some very severe. There were no complications: convulsions, epistaxis, or, above all, broncho-pneumonia. Sleep, appetite, and general well-being were quickly restored. No harmful effects whatever resulted from the treatment. The ozone was produced from a high-frequency apparatus with condenser, but the authors do not state exactly how they ensure the inhalation of the ozone so produced. Their results as reported are undoubtedly remarkable, and show a much greater efficacy for ozonic treatment than any other known therapeutic measure or drug, and they are especially interesting in view of the fact that a sojourn to the seaside frequently ensures a speedy disappearance of the disease. A further interesting point reported by these authors is that radioscopic examination of their patients revealed an abnormal hypertroply of the bronchial and mediastinal glands, and they suggest that in view of this fact a possible tubercular infection as a consequence of the disease is not surprising. The question has been raised whether the spasms are produced by pressure of the enlarged glands on the superior laryngeal nerve, or are a result of irritation of the respiratory mucous membrane. As, however, these authors find that the glands remain enlarged after the spasms have disappeared, the latter hypothesis would appear to be the correct one.

INFLAMMATION of the Thyroid Body secondary to infection at some other point is of relatively common occurrence, but a case reported by Weber in *La Revue Médicale de la Suisse Romande*, in which an acute thyroiditis without suppuration occurred, is of interest because of its rarity. No primary focus of disease could be discovered anywhere in the patient, so that the inflammation would seem to have been idiopathic. The onset of the malady was very sudden with intense and prolonged initial rigors. In twenty-four hours the right lobe of the thyroid swelled to such an extent as to show beneath the skin as a lump the size of a hen's egg. Dysphagia, breathlessness, and neuralgic pains in the ear and the maxilla of the same side, were concomitant symptoms. During the following fortnight a slight diminution in volume of the right lobe was noticed, but at the same time the left lobe increased daily in size and became tender. During the fifth week of the disease the ishmus swelled, and the general condition of the patient grew worse. Improvement in the local condition was very slow, and it was not until the end of the ninth week that the condition was completely cured.

